# Vitamin D Binding Protein Genotype Is Associated with Serum 25-Hydroxyvitamin D and PTH Concentrations, as Well as Bone Health in Children and Adolescents in Finland

**DOI:** 10.1371/journal.pone.0087292

**Published:** 2014-01-30

**Authors:** Minna Pekkinen, Elisa Saarnio, Heli T. Viljakainen, Elina Kokkonen, Jette Jakobsen, Kevin Cashman, Outi Mäkitie, Christel Lamberg-Allardt

**Affiliations:** 1 Department of Food and Environmental Sciences, University of Helsinki, Helsinki, Finland; 2 Folkhälsan Institute of Genetics, Folkhälsan Research Center, Helsinki, Finland; 3 Children’s Hospital, Helsinki University Central Hospital and University of Helsinki, Helsinki, Finland; 4 Division of Nutrition, National Food Institute, Technical University of Denmark, Soborg, Denmark; 5 Department of Food and Nutritional Sciences, University College, Cork, Ireland; Oklahoma State University, United States of America

## Abstract

Vitamin D binding protein (DBP)/group-specific component (Gc), correlates positively with serum vitamin D metabolites, and phenotype influences serum 25-hydroxyvitamin D (S-25(OH)D) concentration. The protein isoform has been associated with decreased bone mineral density (BMD) and increased fracture risk. We examined the role of *GC* genotypes in S-25(OH)D status and BMD in 231 Finnish children and adolescents aged 7−19 yr. BMD was measured with DXA from lumbar spine (LS), total hip, and whole body, and for 175 subjects, radial volumetric BMD was measured with pQCT. Background characteristic and total dietary intakes of vitamin D and calcium were collected. The concentrations of 25(OH)D, parathyroid hormone (PTH), calcium and other markers of calcium homeostasis were determined from blood and urine. Genotyping was based on single-nucleotide polymorphism (rs4588) in the *GC* gene. The genotype distribution was: *GC 1/1* 68%, G*C 1/2* 26% and *GC 2/2* 6%. A significant difference emerged in 25(OH)D and PTH concentrations between the genotypes, (p = 0.001 and 0.028 respectively, ANCOVA). There was also a linear trend in: Gc 2/2 had the lowest 25(OH)D and PTH concentrations (p = 0.025 and 0.012, respectively). Total hip bone mineral content was associated with *GC* genotype (BMC) (p = 0.05, ANCOVA) in boys. In regression analysis, after adjusting for relevant covariates, *GC* genotype was associated with LS BMC and strength and strain index (SSI) Z-score in both genders, and LS BMD in boys. In conclusion, the present study demonstrates the association between *GC* genotypes and S-25(OH)D and PTH concentrations. The results show the influence of DBP genetic variation on bone mass accrual in adolescence.

## Introduction

Low bone mass during adolescence is a risk factor for the development of osteoporosis in later stages of life [1]. Vitamin D deficiency can have a negative effect on bone remodeling and mineralization [2], [3]. Inadequate sun light exposure and nutrition as well as genetic determinants, such as polymorphism of 7-dehydrocholesterol reductase, hepatic microsomal enzyme, and vitamin D binding protein (DBP), also known as group-specific component (Gc), is associated with vitamin D insufficiency [4]. Serum 25-hydroxyvitamin D [S-25(OH)D] is considered to be the most reliable marker of vitamin D status. S-25(OH)D concentrations are partly genetically determined [5]–[7]. A negative correlation exists between S-25(OH)D and serum parathyroid hormone (S-PTH) concentration [8]. PTH is a strong regulator of bone resorption.

For the normal function of vitamin D in the body, most of the vitamin D and its metabolites in blood are bound to DBP. DBP is a polymorphic protein and has a structure related to albumin and the α-fetoprotein gene family [9]. DBP transports 25(OH)D from the liver to the kidneys and other organs to be further converted to the biologically most active vitamin D form, 1,25-dihydroxy-vitamin D [1,25(OH)_2_D]. DBP binds 85 to 90% of the total circulating 25(OH)D and 85% of the total circulating 1,25(OH)_2_D [10]–[11]. Vitamin D can also bind to albumin or chylomicrons at lower levels [12]. There are six common phenotypes of DBP: Gc 1S/1S, Gc 1S/2, Gc 1F/1F, Gc 1S/1F, Gc 1F/2, and Gc 2/2, which differ according to their amino acid composition and glycosylation [13]. Polymorphic DBP differs in its ability to bind vitamin D metabolites, and the phenotype determines plasma concentrations of 25(OH)D and 1,25(OH)_2_D [14]–[17]. Allele frequencies of the *GC* gene vary depending on the ancestral background [17]–[18].

In addition to acting as a transport molecule for vitamin D, DBP has several other important biological roles such as extracellular actin scavenging, leukocyte C5a-mediated chemotaxis, macrophage activation, stimulation of osteoclasts, and transportation of fatty acids [9], [12]–[13], [19]. DBP may impact on bone in two different ways: through binding and transportation of vitamin D metabolites and by activating osteoclasts, as DBP-macrophage activating factor (Gc-MAF) [20]. This factor is thought to be capable of activating osteoclasts by a cellular feedback mechanism that downregulates osteoclast activity when extracellular calcium concentrations increase [21]. Gc-MAF can also stimulate bone resorption by influencing osteoclast calcium sensing.

DBP phenotypes are related to bone fracture risk in postmenopausal women [22] and *GC* genotypes to compression strength index in Caucasian men [23]. Thus, certain phenotypes may be additional risk factors for vitamin D insufficiency and could increase the risk of vitamin D insufficiency-related disease by reducing cell and tissue responses to vitamin D. In addition, the immunological or other unknown functions of DBP could independently affect cell and tissue responses [22], [24].

The impact of the *GC* genotype on vitamin D status and bone mineralization at a young age has received little attention in research. In this study, we investigated the association between vitamin D binding protein genotypes (rs4588) and bone health in children and adolescents. Further, we explored how vitamin D status and *GC* genotypes are associated with bone mineral density (BMD), bone mineral content (BMC), bone structure and the stress and strain index (SSI). We also show for the first time the association between *GC* genotypes and PTH concentrations.

## Materials and Methods

### Ethics Statement

Ethics Committee approval for this study was obtained at the Helsinki University Hospital, Helsinki. The participants and their parents gave an informed written consent before entering the study.

### Study Population

A total of 231 children and adolescents, 160 girls (mean age 13±2.5 years) and 71 boys (12.6±2.7 years), from two different groups [25], [26] were included in this school-based cross-sectional study in the capital region of Helsinki (60°N), southern Finland. The subjects were recruited from primary and secondary schools to cover all age groups. Over 99% of the subjects were Caucasian. Participants and their parents gave an informed written consent before entering the study, which was in accord with the Helsinki Declaration.

### Clinical Data

The subjects together with their parents completed a questionnaire on medical and fracture history, medications, overall health, age at menarche, use of vitamin D and calcium supplements, and details about their physical activity. The dietary vitamin D and calcium intakes were evaluated using a food frequency questionnaire covering over 70 foods [3]. Calculations of food nutrient contents were performed using the Finnish National Food Composition Database, Fineli® (version 2001), which is maintained by the National Institute for Health and Welfare. Physical activity included regular everyday activities (e.g. walking to school), activity at school, and both guided and unguided leisure-time activities. A total physical activity score was obtained by scoring a whole week’s different activities and summing them as described in detail previously [26]. Forms were checked by the researchers, and, if needed, information was clarified by interview.

Height (cm) and weight (kg) were measured and compared with Finnish growth charts. Weight was also expressed as height-adjusted values, as percentages of the mean in a normal population of the same sex and height, according to Finnish standards [27], [28].

Based on questionnaire data and serum gonadotrophin and sex steroid concentrations, pubertal development was scored either as pre-, mid-, or postpubertal (group 1) by a pediatric endocrinologist (OM) or according to Tanner stages I–V (group 2). The Tanner stages were then transformed into pre-, mid-, or postpubertal categories to obtain a united scale for all subjects. Tanner stages I–II were considered as prepubertal, stages III and IV midpubertal and stage V as postpubertal.

### Genotype Analysis

Genomic DNA was isolated from EDTA whole-blood or saliva samples using the Gentra Puregene Kit (Qiagen GmgH, Hilden, Germany) or Wizard® Genomic DNA Purification kit (Promega, Madison, WI, USA). The The rs4588 and (rs7041) SNPs were genotyped with RT-PCR (Mx3000P, Stratagene, La Jolla, CA, USA) using 5′GGC AAA GTC TGA GCG CTT GTT A 3′ and 5′CAG ACT GGC AGA GCG ACT AAA AG 3′ primers and 5′NEP/TTG CCT GAG GCC ACA CC/NEP-3′, 5′MAR/TTG CCT GAT GCC ACA CCC/MAR-3′, 5′NEP/CCA CAC CCA AGG AAC TGG C/NEP-3′, and 5′MAR/CAC ACC CAC GGA ACT GGC/MAR-3′ probes (AllGloTM, Allelogic Biosciences Corporation, Hayward, CA, USA). RT-PCR reactions were carried out in a 20-µL volume using 75 ng template DNA, 500 nM/primer, 250–500 nM/probe, and 1xBrilliant II QPCR Master mix (Stratagene, La Jolla, CA, USA). Amplification was obtained by denaturing at 95°C for 10 min, followed by 55 cycles of denaturing at 95°C for 30 s, annealing at 60°C for 1 min, and extension at 72°C for 30 s. The genotyping results were analyzed by end-point reading in the MxPro software (Mx3000P, Stratagene, La Jolla, CA, USA). Five percent random samples were regenotyped to check for genotyping errors.

### Biochemistry

Blood samples and second void urine samples were collected at 8–10 am after an overnight fast between November and March (group 1) or September and March (group 2). S-25(OH)D was assayed with high-performance liquid chromatography (HPLC, evaluated Vitamin D External Quality Assessment Scheme, DEQAS) in the Central Laboratory of Helsinki University Central Hospital (group 1) or at the Danish Institute for Food and Veterinary Research (subgroup 2). P−/S- serum intact PTH was measured with an immunoluminometric method (group 1) or with a commercial two-site immunoenzymometric assay (group 2) (IEMA; OCTEIA; IDS, Boldon, UK). Plasma/serum concentrations of calcium (P−/S- Ca), urinary Ca (U-Ca), urinary phosphorus (U-Pi), and creatinine (U-Crea) were measured using standard methods.

### Bone Densitometry and pQCT

Bone mineral density (BMD), bone mineral content (BMC), and bone area were measured with DXA (Hologic Discovery A, pediatric software, version 12.4) from the lumbar spine (LS) (L1–L4), total hip, and whole body (WB). Volumetric BMD and bone geometry were measured from the non-dominant radius with pQCT (XCT-2000; Stratec, Pforzheim, Germany) in a group of 175 subjects (104 girls and 71 boys, part of group 1), at the 4% and 66% proximally from the distal end of the non-dominant radius as described in detail previously [29]. The scans were analyzed using version 5.50 of the manufacturer’s software package in which the outer contour of bone is defined with threshold of 280 mg/cm^3^. The scans were analysed using contour mode 2 (45%) and peel mode 1 to assess total bone (TB) and trabecular bone (Trab) parameters at the 4% site. At the 66% site, cortical bone (Cort) was detected with separation mode 1 and a threshold of 710 mg/cm^3^. Also BMC, total bone (TB), cross-sectional area (CSA), and SSI were calculated. Measured values were transformed into Z-scores using equipment-specific age- and sex-adjusted reference data for US Caucasian children in DXA, and in pQCT according to Rauch *et al.* [30]–[31].

### Statistical Analysis

**Figure 1:**
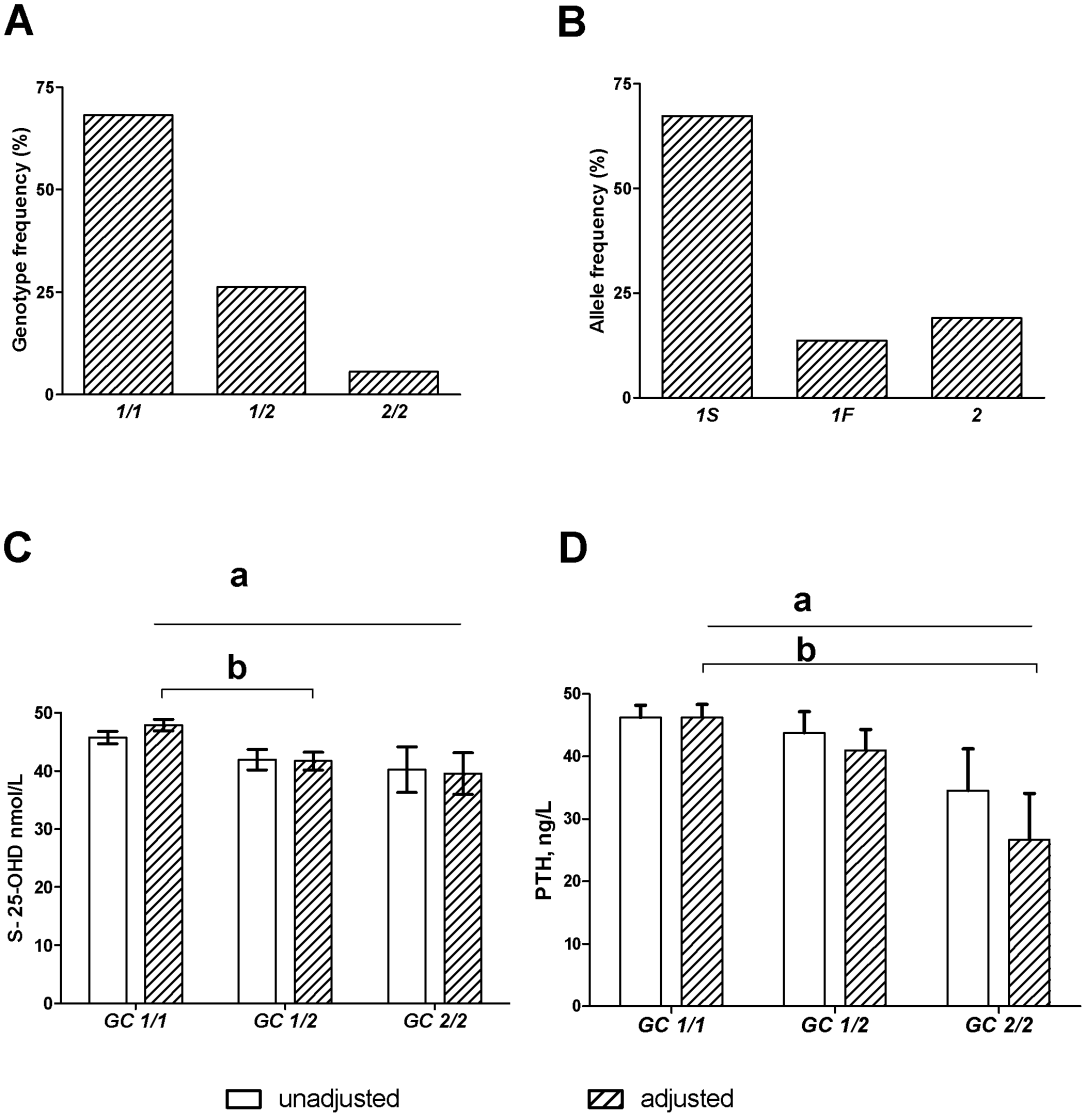
The distribution of *GC* genotypes and alleles in the study population, and associations among 25(OH)D, PTH and *GC* genotypes. (*A*) Distribution of *GC* genotypes *1/1*, *1/2* and *2/2* among the subjects (N = 159, 61 and 13 respectively). *B*) Distribution of *GC* alleles among the subjects (N = 187 and 44 respectively). (*C*) Association of *GC* genotype with S-25(OH)D concentrations. Results are shown for mean (± SE) unadjusted values (ANOVA; p = 0.102, N = 158, 59 and 12, respectively) and mean (± SE) values after adjustment for vitamin D intake, PTH, group 1 or 2, and month when blood sample was taken (ANCOVA; (*a*) p = 0.001, N = 154, 59 and 12). S-25(OH)D concentration was highest in *GC 1/1* and lowest in *GC2/2.* A significant difference were present in S-25(OH)D concentrations between *GC* 1/1 and *GC* 1/2 (ANCOVA; (*b*) p = 0.003). There was a negative linear trend between the genotypes (p = 0.025). (*D*) Association of *GC* genotype with PTH concentrations in group 1. Results are shown for mean (± SE) unadjusted values (ANOVA; p = 0.227, N = 125, 42 and 11 respectively) and mean (± SE) values after adjustment for calcium intake, S-25(OH)D, and month when blood sample was taken (ANCOVA; (*a*), p = 0.028, N = 98, 37 and 8 respectively). PTH levels were highest in *GC 1/1* and lowest in *GC 2/2 (*ANCOVA; *(b)* p = 0.036*).* There was a negative linear trend among the genotypes (ANCOVA; p = 0.012).

Descriptive data are reported as means ± SD. We used a Chi-square test to assess whether DBP genotype distributions were in Hardy–Weinberg equilibrium. Association of variables was tested with Pearson correlation. Logarithmic transformation was applied to non-Gaussian variables. If outliers were detected, Spearman correlation was used instead. Partial correlation was used to illustrate the association after controlling for confounding factor(s). Associations between *GC* genotype, S-25(OH)D and PTH were analyzed with analysis of covariance (ANCOVA), with relevant covariates (shown in Fig. 1*C and 1D* legend texts, respectively). Test for linear trend was performed by using contrast analysis. Before multiple linear regression analysis, several variables were log-transformed to obtain normal distribution (e.g. PTH). Simple regression analysis in the whole study population was first performed to screen potential predictors for BMC, BMD, and a group of 181 subjects (110 girls and 71 boys, group 1) for SSI, and a multivariate linear regression model was used to identify and determine significant predictors for bone mineral content, mass, and SSI. In regression analysis, *GC* genotype was used as a dummy variable [grouped into 0 (*GC 1/1*) and 1 (*GC 1/2*, *GC 2/2*)]. All calculations were performed using PASW 18.0 for Windows. A p-value equal to or less than 0.05 was considered significant.

## Results

### Genotype and Allele Distributions

We identified six different *GC* diplotypes : *1S/1S, 1S/2, 1F/1F, 1F/2, 1S/1F,* and *2/2.* Allele frequencies are shown in Fig. *1B* *.* Because of the small number of subjects in some of the diplotypes, we used only the data of rs4588 genotypes, GC *1/1*, *GC 1/2* and *GC 2/2* in this study. The most common *GC* genotype was *1/1* (68%) and the rarest *2/2* (6%) (Fig. 1*A*). The allele frequency for allele 1 was 81% and for allele 2 19%. The distributions of the rs4588 *GC* genotypes were in compliance with Hardy–Weinberg equilibrium (p = 1.000). The genotypes were equally distributed in both groups.

### Characteristics of Subjects

The background characteristics of the subjects are presented in table 1. Relative weight (height-adjusted weight) was above 20% ( =  overweight) in 31 subjects (15%) and above 40% ( =  obesity) in 7 subjects (3%).

**Table 1:** Baseline characteristics by sex and Gc genotype the comparison are made by ANOVA without covariates.

	*Girls*				*Boys*			
Genotype	*GC 1/1*	*GC 1/2*	*GC 2/2*	P-value	*GC 1/1*	*GC 1/2*	*GC 2/2*	P-value
	N = 105	N = 46	N = 9		N = 54	*N = 15*	*N = 4*	
Age (year)	*12.9±2.3*	*13.0±2.6*	*14.3±3.9*	0.258	*12.61±2.7*	*12.63±2.2*	*12.6±4.8*	0.999
Height (cm)	154.8±11.2	154.3±10.9	153.0±12.5	0.881	154.8±17.6	155.8±17.4	157.7±29.4	0.946
Weight (kg)	46.3±12.3	47.1±12.2	42.9±10.9	0.637	48.9±18.7	48.6±18.2	45.3±22.3	0.931
Weight (%)	4.66±17	7.95±15.6	−1.11±9.0	0.267	8.17±14.5	9±28.1	−7.33±0.6	0.345
Pubertal stage								
prepubertal	34	15	4	0.798	27	9	2	0.608
pubertal	35	15	0		9	3	0	
postpubertal	36	16	5		18	3	2	
Vitamin D intake (µg/day)	8.3±4.7	8.1±5.6	9.7±6.9	0.739	11.1±5.4	12.9±5.7	8.3±2.0	0.427
Ca intake (mg/day)	1486±622	1328±597	1266±509	0.239	1602±528	1638±456	955±125	0.206
S-25(OH)D (nmol/L)	46.0±15.4	42.3±12.4	38.5±9.0	0.153	45.1±12.8	40.7±9.5	45.3±8.0	0.459
S−/P-PTH (ng/L)	43.9±22.4	39.1±13.0	32.0±8.5	0.124	41.6±24.2	45.9±17.8	40±18.3	0.790
S−/P-Ca (mmol/L)	2.40±0.17	2.44±0.20	2.35±0.12	0.316	2.34±0.09	2.31±0.09	2.3±0.03	0.373
S/P-Pi (mmol/L)	1.37±0.18	1.38±0.19	1.29±0.14	0.434	1.42±0.22	1.55±0.12	1.45±0.07	0.090
U-Ca/UCrea (mmol/mmol)	0.21±0.18	0.20±0.2	0.12±0.06	0.510	0.19±0.16	0.25±0.23	0.22±0.15	0.467
U-Pi/UCrea (mmol/mmol)	1.55±0.72	1.56±0.74	1.54±0.47	0.989	2.19±2.7	1.91±0.99	2.3±0.53	0.908
Physical activity score	16.5±4.1	15.9±3.8	13.7±4.3	0.202	14.8±3.6	15.4±4.1	14.5±2.3	0.870

Mean ± SD, N in parentheses.

In girls, were 33% prepubertal, 31% pubertal and, 36% postpubertal. In boys, the corresponding distributions were 52%, 16% and 32%.The mean total intakes of calcium and vitamin D were in line with recommendations [32] in both genders. The mean vitamin D intakes were 8.3 µg and 11.3 µg in girls and boys, respectively. No significant difference in vitamin D intake was present between the genotypes in both genders. We observed that 67% of the study population had S-25(OH)D concentration below 50 nmol/L (68% of girls, 69% of boys). The participants were physically active: 59% had a physical activity score >14 and 9% >18, indicating more than 1.5 and 2 hours, respectively, of physical activity daily.

### *GC* Genotype and S-25(OH)D and PTH Concentrations

A significant negative correlation existed between S-25(OH)D and month of sampling, the concentrations being higher in late autumn than in early spring (r = −0.184, p = 0.005) and between S-25(OH)D and PTH (r =  −0.166, p = 0.022) after controlling for calcium intake. A significant difference was present in S-25(OH)D concentrations among the genotypes, the mean concentration being highest in *GC 1/1* and lowest in *GC 2/2* (p = 0.001, ANCOVA) after adjusting for relevant covariates (vitamin D intake, PTH, group 1 or 2, and month of sampling) (Fig. 1 *C*). When pairwise comparisons were made, only the difference between *GC 1/1* and *GC 1/2* was significant (p = 0.003). However, there was a negative linear trend (p for trend = 0.025) among the genotypes. The relationship between genotypes and PTH was analysed only in group 1 to avoid the bias of the PTH assays in the two groups. The mean PTH concentrations differed among the genotypes; individuals with GC 1/1, who had on average the highest S-25(OH)D concentration, also had the highest PTH concentration, individuals with GC 1/2, who had on average the intermediate S-25(OH)D concentration, also had intermediate PTH concentration, and individuals with GC 2/2, who had on average the lowest S-25(OH)D concentration, also had the lowest PTH concentration (p = 0.028, linear trend p = 0.012), covariates: calcium intake, S-25(OH)D, and month of sampling (Fig. 1*D*). After removing S-25(OH)D from covariates, the association between GC genotype and PTH remained partly significant (p = 0.072 and linear trend p = 0.026).

### Associations between Bone Variables and *GC* Genotype

A significant difference emerged between boys and girls in BMD measured with DXA (ANCOVA, covariates: age, height, weight, S-25(OH)D, puberty, and exercise). Boys had a larger total hip area (p = 0.012), BMC (p<0.001), and BMD (p<0.001). Girls had larger WB BMD (p = 0.035). Also in volumetric BMD measured with pQCT, boys had a higher BMC in distal and proximal radius (p<0.001 and 0.001, respectively), higher SSI (p<0.001), and larger total bone area in distal and proximal sites (p = 0.006 and p = 0.000, respectively). Boys also had higher cortical BMD (p = 0.001).

BMD values based on DXA measurements between genotypes GC 1 and the combined genotypes GC 1/2 and 2/2 are presented in table 2, 3 and 4 (*ANOVA and ANCOVA*). After adjusting for relevant covariates (age, sex, weight, height, S-25(OH)D, pubertal stage, and physical activity), a significant difference emerged in total hip BMC between genotypes in boys (p = 0.05) (Table 4).

**Table 2:** Bone variables for genotype 1/1 and genotypes Gc 1/2 and 2/2 combined in the whole study population measured with DXA.

Genotype	*GC 1/1*	*GC 1/2 and 2/2*	P_a_
			P_b_
N	158_a_	73_a_	
	123_b_	59_b_	
LS area (cm^2^)	47.5±9.8_a_	46.6±9.8_a_	0.495_a_
	45.7±9.2_b_	45.8±9.8_b_	0.940
LS BMC (g)	38.5±14.8_a_	37.4±15.1_a_	0.576_a_
	36.2±14.0_b_	36.1±15.0_b_	0.993_b_
LS BMD (g/cm^2^)	0.78±0.16_a_	0.77±0.16_a_	0.675_a_
	0.76±0.16_b_	0.76±0.17_b_	0.988_b_
LS BMD Z	0.13±0.98_a_	−0.09±0.93_a_	0.110_a_
	0.12±0.98_b_	0.01±0.95_b_	0.384_b_
Total hip area (cm^2^)	29.2±5.7_a_	28.4±5.6_a_	0.336_a_
	28.1±4.9_b_	28.0±5.6_b_	0.685_b_
Total hip BMC (g)	24.5±7.8_a_	23.5±7.7_a_	0.369_a_
	23.0±6.9_b_	22.9±7.6_b_	0.855_b_
Total hip BMD (g/cm2)	0.82±0.12_a_	0.81±0.14_a_	0.540_a_
	0.81±0.12_b_	0.81±0.14_b_	0.964_b_
Total hip BMD Z	0.22±0.89_a_	0.16±0.9_a_	0.619_a_
	0.27±0.92_b_	0.23±0.9_b_	0.763_b_
WB area (cm^2^)	2140±376_a_	2114±370_a_	0.674_a_
	2066±370_b_	2060±368_b_	0.651_b_
WB BMC (g)	1803±508_a_	1770±500_a_	0.681_a_
	1701±491_b_	1700±504_b_	0.985_b_
WB BMD (g/cm^2^)	0.83±0.10_a_	0.822±0.11_a_	0.714_a_
	0.81±0.10_b_	0.81±0.11_b_	0.945_b_
WB Z	0.086±0.83_a_	−0.085±0.75_a_	0.196_a_
	0.024±0.82_b_	0.109±0.73_b_	0.499_b_

Values are presented as mean ± SD. ANOVA (a) and ANCOVA (b). ANCOVA covariates: Sex and age (except for the Z-score), height, weight, S-25OHD, puberty and physical activity score. LS = lumbar spine, WB = whole body, BMC = bone mineral content, BMD =  bone mineral density, Z = age and sex adjusted value (Z-score).

**Table 3:** Bone variables for genotype 1/1 and genotypes Gc 1/2 and 2/2 combined in girls measured with DXA.

Girls			
Genotype	*GC 1/1*	*GC 1/2* and *2/2*	P_a_
N	105_a_	55_a_	P_b_
	84_b_	44_b_	
LS area (cm^2^)	46.9±8.8_a_	46.9±9.3_a_	0.959_a_
	45.6±8.2_b_	46.3±9.5_b_	0.363_b_
LS BMC (g)	38.2±13.3_a_	38.6±14.9_a_	0.876_a_
	36.4±12.5_b_	37.5±15.4_b_	0.282_b_
LS BMD (g/cm^2^)	0.79±0.15_a_	0.80±0.16_a_	0.801_a_
	0.77±0.15_b_	0.785±0.17_b_	0.406_b_
LS BMD Z	−0.003±0.98_a_	−0.05±1.01_a_	0.762_a_
	0.023±1.04_b_	0.007±1.04_b_	0.923_b_
Total hip area, (cm^2^)	28.4±4.1_a_	28.1±4.5_a_	0.698_a_
	27.8±3.6_b_	28.1±4.6_b_	0.427_b_
Total hip BMC (g)	23.1±5.6_a_	23.1±6.7_a_	0.947_a_
	22.4±5.1_b_	22.9±6.9_b_	0.321_b_
Total hip BMD(g/cm2)	0.81±0.11_a_	0.810±0.14_a_	0.894_a_
	0.80±0.109_b_	0.803±0.147_b_	0.730_b_
Total hip BMD Z	0.23±0.9_a_	0.191±0.96_a_	0.809_a_
	0.29±0.96_b_	0.252±0.94_b_	0.818_b_
N	75_a_	35_a_	
	57_b_	29_b_	
WB a (cm^2^)	2147±320_a_	2143±341_a_	0.951_a_
	2093±321_b_	2095±344_b_	0.888_b_
WB BMC (g)	1791±421_a_	1831±478_a_	0.652_a_
	1722±422_b_	1763±499_b_	0.152_b_
WB BMD (g/cm^2^)	0.82±0.09_a_	0.84±0.11_a_	0.361_a_
	0.81±0.09_b_	0.83±0.12_b_	0.130_b_
WB BMD Z	−0.020±0.79_a_	0.006±0.8_a_	0.881_a_
	−0.042±0.83_b_	0.120±0.75_b_	0.351_b_

Values are presented as mean ± SD. ANOVA (a) and ANCOVA (b). ANCOVA covariates: age (except for the Z-score), height, weight, S-25OHD, puberty and physical activity score. LS = lumbar spine, WB = whole body, BMC = bone mineral content, BMD =  bone mineral density, Z = age and sex adjusted value (Z-score).

**Table 4:** Bone variables for genotype 1/1 and genotypes Gc 1/2 and 2/2 combined in boys measured with DXA.

Boys			
Genotype	*GC 1/1*	*GC 1/2* and *2/2*	P_a_
N	53_a_	18_a_	P_b_
	39_b_	15_b_	
LS area (cm^2^)	48.6±11.6_a_	45.6±11.3_a_	0.344_a_
	46.1±11.3_b_	44.3±11.0_b_	0.212_b_
LS BMC (g)	39.2±17.4_a_	33.7±15.7_a_	0.239_a_
	35.5±16.8_b_	32.2±15.8_b_	0.102_b_
LS BMD (g/cm^2^)	0.77±0.17_a_	0.71±0.15_a_	0.158_a_
	0.74±0.16_b_	0.70±0.15_b_	0.100_b_
LS BMD Z	0.391±0.92_a_	−0.200±67_a_	0.014_a_
	0.358±0.81_b_	−0.07±0.66_b_	0.067_b_
Total hip area, (cm^2^)	30.7±7.78_a_	29.2±8.4_a_	0.506_a_
	28.7±7.0_b_	27.4±8.1_b_	0.087_b_
Total hip BMC (g)	27.1±10.5_a_	24.7±10.4_a_	0.412_a_
	24.5±9.6_b_	22.6±9.7_b_	0.050_b_
Total hip BMD (g/cm^2^)	0.86±0.14_a_	0.82±0.13_a_	0.355_a_
	0.83±0.13_b_	0.80±0.12_b_	0.295_b_
Total hip BMD Z	0.21±0.87_a_	0.06±0.68_a_	0.510_a_
	0.28±0.83_b_	0.08±0.74_b_	0.420_b_
WB a (cm^2^)	2130±447_a_	2060±425_a_	0.554_a_
	2025±431_b_	1999±422_b_	0.327_b_
WB BMC (g)	1820±614_a_	1648±527_a_	0.290_a_
	1668±580_b_	1582±518_b_	0.070_b_
WB BMD (g/cm^2^)	0.835±0.123_a_	0.784±0.10_a_	0.117_a_
	0.806±0.12_b_	0.776±0.09_b_	0.088_b_
WB BMD Z	0.23±0.87_a_	−0.26±0.62_a_	0.029_a_
	0.209±0.78_b_	−0.144±0.66_b_	0.111_b_

Values are presented as mean ± SD. ANOVA (a) and ANCOVA (b). ANCOVA covariates: age (except for the Z-score), height, weight, S-25OHD, puberty and physical activity score. LS = lumbar spine, WB = whole body, BMC = bone mineral content, BMD =  bone mineral density, Z = age and sex adjusted value (Z-score).

### Regression Analysis of BMC, BMD, and SSI

To determine the factors associated with LS, total hip, and WB BMC, BMD Z-scores, and SSI Z-scores in children and adolescents, multiple linear regression analyses were performed. In these regression models, age, height, weight, *GC* genotype, S-25(OH)D and PTH concentrations, physical activity, and pubertal stage were included (Tables 5 and 6). The regression model accounted for 81–87% of the variance (adjusted R^2^) in LS BMC, 82% of the variance in total hip BMC, 75–87% of the variance in WB BMC (Table 5), 16–26% of the variance in LS BMD Z-score, 9–24% of the variance in total hip BMD, 14–19% of the variance in WB BMD Z-score, and 8–26 of the variance in SSI Z-score (Table 6). Among all the independent variables; weight, age, pubertal stage and *GC* genotype were the significant determinants of LS BMC in both genders, and in addition height was a significant determinant of LS BMC in girls (Table 5).

**Table 5:** Linear regression analysis for determinants of bone mineral content in groups defined by sex.

	LSBMC				Total hip BMC				WB BMC		
	*r^2^*	*ß*	*P*		*r^2^*	*ß*	*P*		*r^2^*	*ß*	*P*
***Girls*** (N = 159)											
Regression model	**0.873**			**≤0.001**	**0.824**			**≤0.001**	**0.748**		**≤0.001**
Height (cm)	**0.003**	0.124		**0.021**	**0.131**	0.248		**≤0.001**		0.006	0.934
Weight (kg)	**0.015**	0.111		**0.016**		0.044		0.417	**0.578**	0.475	**≤0.001**
Pubertal stage	**0.005**	−0.082		**0.014**	**0.011**	−0.126		**0.001**	**0.067**	0.201	**≤0.001**
S- 25(OH)D (nmol/L)	**0.629**	0.522		**≤0.001**	**0.655**	0.565		**≤0.001**	**0.005**	−0.116	**0.031**
*GC* genotype*	**0.003**	0.065		**0.030**	**0.017**	0.105		**0.003**		−0.062	0.144
Exercise (physical activity score)		−0.033		0.255		−0.024		0.480		0.020	0.627
Age	**0.218**	0.357		**≤0.001**	**0.010**	0.179		**0.002**	**0.098**	0.496	**≤0.001**
***Boys*** (N = 73)											
Regression model	**0.812**			**≤0.001**	**0.824**			**≤0.001**	**0.873**		**≤0.001**
Height (cm)		0.001		0.997	**0.045**	0.228		**≤0.001**		0.073	0.563
Weight (kg)	**0.030**	0.269		**0.017**	**0.014**	0.228		**0.032**	**0.061**	0.382	**≤0.001**
Pubertal stage	**0.031**	0.297		**0.003**	**0.015**	0.249		**0.008**	**0.019**	0.188	**0.020**
S-25(OH)D (nmol/L)		0.007		0.894		0.069		0.191		0.084	0.066
*GC* genotype*	**0.010**	−0.019		**0.049**		−0.073		0.161	**0.008**	−0.093	**0.041**
Exercise (physical activity score)		−0.015		0.807		−0.742		0.461		−0.003	0.954
Age	**0.741**	0.416		**0.002**	**0.750**	0.287		**0.019**	**0.785**	0.394	**≤0.001**

*r^2^* values are adjusted and *ß* values are standardized. BMC = bone mineral content, LS =  lumbar spine, WB =  whole body.

* *GC* genotype is used as a dummy variable.

**Table 6:** Linear regression analysis for determinants of lumbar spine, total hip and whole body BMD and strength strain index Z-score in groups defined by sex.

	LS BMD (Z-score)			Total hip BMD (Z-score)			WB (Z-score)			SSI (Z-score)		
	r^2^	ß	P	r^2^	ß	P	r^2^	ß	P	r^2^	ß	P
***Girls*** (N = 159)										(N = 73)		
Regression model	**0.260**		**≤0.001**	**0.237**		**≤0.001**	**0.143**		**0.003**	**0.265**		**≤0.001**
Height (cm)	**0.097**	−0.503	**≤0.001**	**0.232**	−0.654	**≤0.001**	**0.064**	−0.506	**0.002**		−0.668	0.060
Weight (kg)	**0.163**	0.797	**≤0.001**	**0.005**	0.739	**≤0.001**		0.245	0.110	**0.104**	0.525	**0.003**
Pubertal stage		0.080	0.276		0.066	0.413		0.093	0.407		0.241	0.060
S-25(OH)D (nmol/L)		0.025	0.769		−0.906	0.339	**0.079**	0.451	**≤0.001**	**0.051**	−0.529	**0.010**
*GC* genotype*		−0.077	0.276		−0.050	0.482		−0.046	0.645	**0.028**	−0.272	**0.011**
Exercise (physical activity score)		0.037	0.532		0.073	0.303		0.010	0.921		0.424	0.086
PTH										**0.082**	0.603	**0.005**
***Boys*** ** (**N = 73)				N = 56				N = 73		N = 42		
Regression model	**0.162**		**0.018**	**0.090**		**0.015**	**0.195**		**0.015**	**0.082**		0.187
Height (cm)	**0.039**	−0.540	**0.042**		−0.478	0.198		−0.023	0.940		−0.728	0.124
Weight (kg)	**0.056**	0.659	**0.006**	**0.090**	4.474	**0.006**		−0.113	0.663		0.654	0.097
Pubertal stage		0.001	0.991		−0.191	0.477		0.102	0.616	**0.067**	0.536	**0.043**
S-25(OH)D (nmol/L)		−0.017	0.885		0.081	0.564		0.219	0.067		−0.008	0.962
*GC* genotype*	**0.067**	−0.243	**0.039**		−0.138	0.335	**0.049**	−0.252	**0.053**		0.004	0.982
Exercise (physical activity score)		0.001	0.991		0.091	0.584	**0.146**	0.356	**0.023**	**0.015**	0.431	**0.018**
PTH											−0.033	0.833

*r^2^* values are adjusted and ß values are standardized. PTH was included in SSI model. BMD = bone mineral density, LS =  lumbar spine, WB =  whole body, SSI =  strength strain index, * GC genotype is used as a dummy variable.

In the regression model for LS BMC, the *GC* genotype variable was a significant determinant in both genders (p = 0.030, p = 0.049, Table 5). Weight, pubertal stage and age in boys, and height, S-25(OH)D concentration, pubertal stage, age and *GC* genotype in girls, were significant determinants of total hip BMC. Weight, pubertal stage, and age in boys and weight, pubertal stage, S-25(OH)D concentration and age in girls determined WB BMC (Table 5). Determinants for LS BMD Z-score were height and weight in girls and weight and *GC* genotype in boys; whereas height and weight in girls and weight in boys were the significant determinants for total hip BMD Z-scores.

Height and S-25(OH)D concentration in girls and physical activity in boys were the significant determinants of WB BMD Z-score. In addition *GC* genotype was almost a significant determinant for WB BMD Z-score in boys (p = 0.053). Weight, S-25(OH)D concentration, *GC* genotype and PTH in girls determined SSI. In boys, no significant regression model was found for SSI (N = 42, p = 0.187, Table 6). Because of this, regression analysis was also performed in the total study population with the same determinants as in Table 6; in this regression model, weight (p = 0.005), S-25(OH)D concentration (p = 0.013), *GC* genotype (3.1% of the variance, p = 0.015), and physical activity (p = 0.001) were significant determinants of SSI Z-score.

## Discussion

We have studied for the first time vitamin D binding protein polymorphism among healthy school-children and adolescents in Finland. We found that the genotypes were in Hardy and Weinberg equilibrium and that the distribution differed from the Dutch and Danish populations. In our study population, the frequency of the rare *GC 2* allele was 19%, in the Dutch population 27% [33], and in the Danish population 26% [14]. The special genetic origin and long isolation of Finns may explain the different frequencies of alleles relative to other European countries [18], [34]–[35].

Our study revealed a significant difference in 25(OH)D concentrations among the GC genotypes in children and adolescents. This finding is in line with several other studies [4], [33], [36]–[39]. We observed that the S-25(OH)D concentrations were highest in subjects who had genotype *GC 1/1*, intermediate in *GC 1/2* and lowest in *GC 2/2*. Similar finding has been shown in other studies [9], [15]. It has been proposed that the Gc2 protein has significantly lower affinity to 25(OH)D compared to Gc1S and Gc1F proteins [16]. Lauridsen [15] showed that plasma concentration of DBP was significantly higher in phenotype 1/1, intermediate in 1/2 and lowest in 2/2.They speculated that the observed difference could be due to different characteristics of the Gc phenotypes. We did not measure the DBP concentrations in the present study.

The relationship between genotypes and PTH was analysed only in group 1 to avoid the bias of the two assays. PTH concentration correlated negatively with S-25(OH)D and similar finding was present in the whole population. This correlation has been shown in many studies [8]. Interestingly, however, within the genotype groups, *GC 2/2*, with the lowest 25(OH)D concentrations, also had the lowest PTH concentration, and *GC 1/1*, with the highest 25(OH)D concentrations, had the highest PTH concentration (group 1). Taes *et al.* [40] have noted a similar association between DBP phenotype and S- PTH concentrations in elderly men, but this finding was not statistically significant. In addition, it is noteworthy that within the genotypes an inverse correlation existed between PTH and 25(OH)D. The distribution of the subjects from the different groups was similar in all *GC* groups. Hence, some mechanism associated with *GC* could underlie these results.

Only a few studies have investigated vitamin D binding protein polymorphism and bone health [22]–[23], [33], [41]–[43]. To the best of our knowledge, this study is the first to evaluate the association between *GC* genotype and bone health in children and adolescents. One strength of our study is the extensive background characteristic data we have collected, i.e. sunshine exposure, dietary intakes of vitamin D and calcium as well as intake from supplements and physical activity, which enables the use of more specific covariates and increases the power of statistical analysis. We showed that total hip BMC was lower in the combined Gc 1/2 and Gc 2/2 genotype in boys. In regression models, LS BMD Z-scores (p = 0.039) and WB BMD Z-scores (p = 0.053) were associated with *GC* genotype in boys. In girls, by contrast, we detected no association between *GC* genotype and BMD. Papiha *et al*. [43] have reported a relationship between *GC* gene (TAAA)(n)-Alu polymorphism and spinal as well as total hip BMD in men. In addition, DBP polymorphism has been shown to be associated with BMD and the fracture risk in men [42]. In a study on Japanese postmenopausal women, SNPs linkage disequilibrium with DBP was associated with radial BMD [41]. Lauridsen *et al.* [22] found no significant difference in BMD in postmenopausal women among Gc phenotypes. Giroux *et al.* [44] evaluated the association of 23 candidate genes with high-density polymorphisms with BMD in premenopausal Canadian women. The genes studied were involved in calcium and vitamin D regulation or associated with estrogen metabolism and bone. One of the genes studied was the DBP coding gene, but no association was found with BMD. However, genes involved in the biosynthesis of vitamin D (CYP2R1 and CYP27A1) were associated with BMD.

In our study, an association existed between *GC* genotype and a marker of bone strength, SSI [45]. Low SSI, vBMD, cortical area and aBMD have been reported to associate with increased fracture risk in children [46]. Previous findings in adults have reported an association between DBP haplotype and fracture risk, especially when dietary calcium intake was low [33]. In our study population, the mean calcium intake was high, but we nevertheless found a similar association between genotype and SSI. Xu *et al.* [23] established an association between compression strength index (CSI) and DBP polymorphism in Caucasian men. Lauridsen *et al^.^* [22] observed a difference in the fracture risk between genotypes in postmenopausal women; the risk was highest in *GC 1/1*, intermediate in *GC 1/2*, and lowest in *GC 2/2*. In our study, the SSI was lowest in *GC2/2*, intermediate in *GC 1/1* and highest in *GC 1/2.* These results resemble previous findings on the association of *GC* gene polymorphism and bone [22]–[23], [33], but the number of subjects in our study was low and no firm conclusions can be drawn. However, when the heterozygote and *GC 2/2* groups were combined in regression analysis, the group size was larger and a similar association was noted. These findings may illustrate a difference in the genetic effect of DBP between young developing bone and the elderly skeleton. The rs4588 (and rs7041) polymorphism, which we studied, is in exon 11 (domain III), an area responsible for non-sterol activities together with domain II and also the macrophage/osteoclast activation of DBP [33]. The difference in bone fractures has been proposed to be due to Gc-MAF formation [22]. In individuals with genotype *GC 1/1*, the concentration of DBP is higher, and therefore, there is more substrate for Gc-MAF formation. Furthermore, the rate of osteoclast formation is higher. Another reason could be differences in glycosylation of DBP protein between the genotypes because the formation of Gc-MAF requires the removal of glucose residuals.

We found that PTH concentrations were higher in those genotype groups where 25(OH)D was also higher. Lauridsen *et al.* [15] found no difference in PTH between the genotype groups, although the genotypes differed in 25(OH)D concentrations. In some studies concerning DBP, PTH has not been measured. Carpenter *et al.* [17] observed no effect of *GC* genotype on the relationship with 25(OH)D and PTH, but did not study the relationship among *GC* genotype and PTH. It has been shown that vitamin D prohormones directly suppress the secretion of PTH from the parathyroid glands, and this suppression requires the vitamin D receptor [47]. According to our results, PTH was low in genotype *2/2*, in which the S-25(OH)D concentration was also low. We hypothesize that in this case the amount of free (unbound) 25(OH)D and 1,25(OH)D is higher in genotype *2/2*, and thus, there are more free forms available to suppress PTH secretion from the glands. Powe *et al.* [48] hypothesized that circulating DBP is an inhibitor of the biological action of vitamin D in humans. The inverse relationship that we discovered between *GC* genotypes and BMD could be caused by the difference in free or bound serum DBP levels among different genotypes, which can lead to differences in the inhibition of vitamin D action and in osteoclast function by Gc-MAF activation.

A weakness of our study is the limited number of subjects, especially in the rarest genotype *GC 2/2*, and the small number of boys. Another limitation is that the data consist of two groups. Because of the low frequency of the rarest genotype, it was not possible to divide subjects into groups according to puberty. In girls, the distribution was quite even, but only 16% of the boys were pubertal. When the subjects were grouped according puberty and genotype, girls had fairly even group sizes, except for the *GC 2/2* group, where girls were either prepubertal or postpubertal and none were in puberty. Boys had no pubertal subjects in the genotype group *GC 2/2*. However, when the genotypes *GC* 1/2 and 2/2 were combined in the analyses, the distribution into puberty groups was more even. The observational design of our study prevented us from establishing a causal relationship between *GC* genotype, S-25(OH)D, PTH and bone accrual in adolescence.

## Conclusion

This is the first study to examine the association between *GC* genotype on bone health in children and adolescents. These results suggest that S-25(OH)D and PTH concentrations differ among *GC* genotypes and that variation exists in BMD and bone strength index among genotypes. Variation in S-25(OH)D among genotypes suggests differences in vitamin D utilization. BMD variables among the genotypes differed only in boys. This may be due to gender differences in the phase of skeletal development. We conclude that DBP may be one factor affecting bone mass accrual during adolescence. In the future, DBP could be useful in detecting individuals who are more susceptible to vitamin D deficiency, especially while their skeleton is still developing.
